# Decontamination of Fused-Silica Surfaces by UVC Irradiation as Potential Application on Touchscreens

**DOI:** 10.3390/microorganisms12102099

**Published:** 2024-10-21

**Authors:** Ben Sicks, Oksana Gurow, Florian Sommerfeld, Martin Hessling

**Affiliations:** Institute of Medical Engineering and Mechatronics, Ulm University of Applied Sciences, 89075 Ulm, Germany; ben.sicks@thu.de (B.S.); oksana.gurow@thu.de (O.G.); florian-sommerfeld@web.de (F.S.)

**Keywords:** UVC-irradiation, surface contamination, surface disinfection, *Staphylococcus carnosus*, *Escherichia coli*, *Acinetobacter kookii*, *Enterococcus mundtii*, *Micrococcus luteus*, *Pseudomonas stutzeri*

## Abstract

The contamination of surfaces by antibiotic-resistant pathogens presents an escalating challenge, especially on touchscreens in public settings such as hospitals, airports, and means of transport. Traditional chemical cleaning agents are often ineffective and leave behind harmful residues. Thus, the application of optical radiation is gaining relevance as a rapid, effective, and environmentally friendly disinfection method. This study examines the contamination of publicly accessible touchscreens and the efficacy of an irradiation approach for the radiation disinfection of microorganisms on quartz surfaces with UVC LEDs. In this setup, the LED radiation is laterally coupled into a quartz plate that serves as cover glass of a simplified touchscreen model. The process allows for the irradiation of microorganisms on the surface, without the user being exposed to hazardous radiation. To assess the efficacy of the disinfection process, a range of bacteria, mostly ESKAPE surrogates, such as *Staphylococcus carnosus*, *Acinetobacter kookii*, *Escherichia coli*, *Enterococcus mundtii*, and additionally *Micrococcus luteus*, were spread over a quartz plate with a homebuilt nebulization system. After operating the side-mounted LEDs for 30 s, a reduction in all bacteria except *M. luteus* by more than three orders of magnitude was observed. In the case of *M. luteus*, a significant reduction was achieved after 60 s (*p* < 0.05). This result demonstrates the potential of side-mounted UVC LEDs for rapid disinfection of touchscreens between two users and thus for reducing the spread of pathogens without irradiating humans.

## 1. Introduction

The disinfection of surfaces plays a crucial role in preventing the transmission of pathogens, particularly in healthcare settings, public transportation, and high-traffic public areas [1,2,3,4,5]. With the rise of antibiotic-resistant bacteria, the need for effective disinfection methods is becoming increasingly urgent [6,7,8,9,10,11,12]. It is crucial to recognise the importance of ESKAPE pathogens in touchscreen contamination. ESKAPE is an acronym used in infectious disease medicine to describe a group of particularly problematic bacterial pathogens. These bacteria are often the cause of nosocomial infections and have developed resistance to many antibiotics and are frequently found on touchscreens even in healthcare settings [1].

Conventional chemical disinfectants exhibit limitations, as they can lead to tolerance development against biocidal active ingredients in antiseptics and disinfectants [13] and pose potential health risks. Against this backdrop, alternative methods, such as disinfection using optical radiation, particularly ultraviolet (UV) radiation [14,15,16,17,18,19,20], have emerged as promising approaches in recent years.

Previous studies have revealed that UV radiation, particularly ultraviolet C (UVC) radiation in the 100 to 280 nm range, can inactivate microorganisms by damaging their DNA and RNA [21]. This prevents their reproduction and function, ultimately leading to their death [22,23,24,25,26,27]. Unfortunately, this property also poses a health risk for humans, limiting its practical application. The European DIRECTIVE 2006/25/EC and the American Conference of Governmental Industrial Hygienists (ACGIH) limit the allowed skin exposure to 3 mJ/cm^2^ over eight hours [28,29]. Given the increasing contamination of frequently touched surfaces, such as touchscreens in public facilities, the need for safe and efficient disinfection methods is particularly high [22,30,31].

Previous work on this topic has explored both UVA (315–400 nm) [32] and Far-UVC (200–240 nm) LEDs [33], both of which were promising approaches but are not yet suitable for practical application. In UVA experiments, the disinfection time lasted several hours, making quick and efficient disinfection impractical in everyday use. Investigations with Far-UVC LEDs exhibited better results, enabling rapid disinfection of a small area of 5 × 5 cm^2^ within 20 s. However, Far-UVC LEDs currently have a limited lifespan of a few hundred hours, further restricting their applicability. Additionally, the area covered in the Far-UVC study was too small for practical use.

A more promising alternative is the application of UVC LEDs in the 250 to 280 nm spectral range. These LEDs are not only more efficient but also have a significantly longer lifespan of up to 10,000 h and are highly cost-effective, priced at approximately 1 USD per LED. This makes their integration into real-world applications, such as touchscreens, both economically and technically feasible.

Another important difference from our previous studies is the investigation of a broader range of microorganisms. While our earlier work focused exclusively on the Gram-positive bacterium *Staphylococcus carnosus*, which has a UV sensitivity similar to *S. aureus*, this is the first study also including Gram-negative bacteria. This significantly expands the scope of disinfection technology and provides a better foundation for practical application.

**Figure 1:**
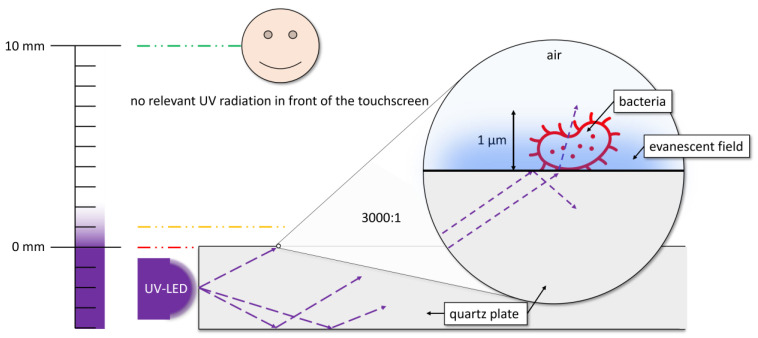
This diagram illustrates the operation of total internal reflection and the impact of evanescent fields and outgoing ray on surface exposure. In this regard, the coupled ultraviolet LED, which employs the quartz plate as a light guide, emits radiation at the contaminated site. The millimetre scale is also provided with a colour scale to illustrate the intensity of the evanescent field depending on the distance to the quartz plate. The differently coloured lines (red = 0 mm, yellow = 1 mm and green = 10 mm) indicate distances to the quartz plate and are intended to represent the rapid decline in radiation exposure. The enlargement to a few micrometres illustrates the irradiation of a bacterium on the quartz surface by outgoing rays and the evanescent field.

This study aims to bridge the gap between previous approaches and real-world applicability by using UVC LEDs placed at the edges of quartz plates for targeted disinfection. Through the use of total internal reflection, the radiation can be directed towards contaminated areas, minimising user exposure. This is achieved by transmitting the radiation through the quartz plate until it is scattered by contamination from dust and touch. Additionally, the evanescent field generated at the interface enhances the disinfection effect on the surface [34,35]. Figure 1 illustrates the aforementioned effects. However, dust particles and the evanescent field can cause some radiation to escape, potentially exposing people to it, which is a potential hazard.

The described approach differs from other methods, such as the use of disinfection benches in which the devices are simply irradiated from above, called UV sterilisers for smart devices, and the simplest but not safest alternative, which is “manual touchscreen disinfection” using a UV hand lamp.

Bacterial suspensions decline when irradiated. A simple exponential decline does not account for all relevant factors. The initial damage to the cell wall, its Gram characterisation, and the repair mechanisms are not taken into account. Using a more accurate model description and flexible evaluation methods would be more advantageous. The novelty of this study lies in the combination of a broader microbial evaluation, the application of cost-effective UVC LEDs, and the use of advanced optical techniques to enable safe and rapid disinfection in real-world environments and devices.

## 2. Materials and Methods

**Figure 2:**
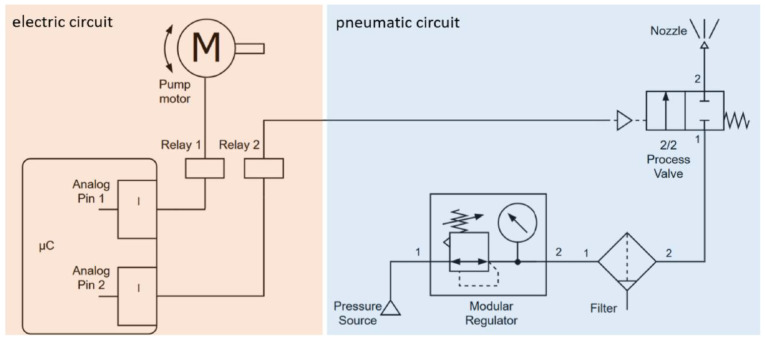
The circuit diagram of the developed bacterial contamination system is divided into two sections: the electrical system (orange) and the pneumatic system (blue). The components of the pneumatic system are identified with the pressure air inlet (1) and the outlet for the air consumer (2). The connection between the electrical and pneumatic schematics is established through the electrical connection for the control signal (triangle with dotted line) of the 2/2-way process valve. The solid lines represent the conventional conductors (electricity/pneumatic).

***Nebulisation system:*** To achieve a uniform contamination of the quartz plates, an ultrasonic nebuliser (SK508, CY Spray, Dongguan, China) was employed with a two-bar air pressure and a microcontroller-controlled peristaltic pump. This home-built system generated small droplets, ensuring even contamination and enhanced adhesion. A schematic system structure with block diagrams is presented in Figure 2. The system was operated for 1.2 s to nebulise 1.75 mL of bacterial suspension to be sprayed, with the aim of achieving sufficient initial concentration on the quartz surface, which was previously cleaned with 70% ethanol. In order to obtain a non-irradiated reference sample, an additional quartz plate was sprayed.

The initial bacterial concentrations of the bacterial suspension to be sprayed were adjusted with the optical density at 600 nm (OD600) with a spectrophotometer (SPECORD 250 PLUS, Analytik Jena, Jena, Germany). At the target OD600 of 0.5, the bacterial concentration was 10^8^ colony-forming units (CFU) per millilitre. This resulted in a surface contamination of 10^6^ CFU/cm^2^.

The distribution of the nebulisation was examined by spraying an ink pen colour pigment and water mixture and subsequent microscopy of a surface area of 0.25 mm^2^. To evaluate the persistence of organisms on the quartz glass plate, control swabs were only taken hours after the test. This shows that the experimental organisms are not affected by environmental conditions. Bacterial reductions are exclusively attributable to irradiation.

**Figure 3:**
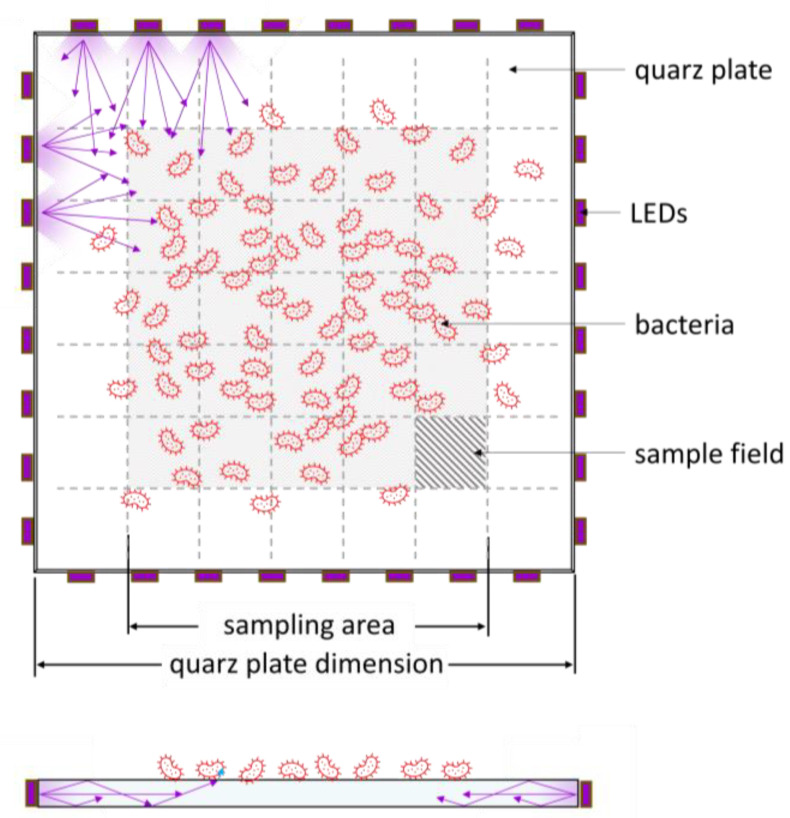
Top and side view of the irradiation arrangement comprising 32 ultraviolet LEDs that surrounded the quartz plate from the side, with a total size of 30 × 30 cm^2^ and emitted radiation with a wavelength of 275 nm. The sampling area was limited to the contaminated surface, which represented a contaminated touchscreen. The sampling area was divided into 25 sampling fields, each with an area of 4 × 4 cm^2^. The violet arrows illustrate the principle of radiation distribution within the light guide.

***Irradiation setup:*** In order to conduct the irradiation experiments, a 30 × 30 × 0.4 cm^3^ quartz plate from GVB (Herzogenrath, Germany) was subjected to contamination and subsequent decontamination through irradiation. This irradiation was conducted via lateral coupling of 32 UVC LEDs (C3535DUVC -QB-Q5-D, Luckylight, Shenzhen, China) with a peak wavelength of 275 nm, arranged around the glass plate and operated with a forward current of 40 mA (Figure 3). An additional contaminated quartz plate served as an unirradiated control.

In order to evaluate the uniformity of the UV irradiance distribution, the irradiance was quantified at a distance of 1 mm above the contaminated quartz surface, due to the geometry of the measuring head. This was achieved by scanning a grid of measurement points with the Optometer X1 of Gigahertz Optik GmbH (Türkenfeld, Germany). The measurements were conducted three times, with the results averaged and normalised. A second series of measurements was conducted at distances ranging from 10 cm to 50 cm to evaluate the potential exposure of the user to radiation. The data obtained enabled the calculation of the maximum duration of skin exposure, thus facilitating the exclusion of potential health risks.

This calculation was based on the threshold limit values of the ACGIH and European DIRECTIVE 2006/25/EC for skin irradiation limit of 3 mJ/cm^2^ over an 8 h period. The equation used for this calculation is given in Equation (1) [28,29].

(1) $$H_{\mathrm{dose}}={\;E}_{\mathrm{irradiance}\;}{\;*\;t}_{\mathrm{exposure}\;\mathrm{time}}$$

The maximum permissible skin exposure times (t_max exposure time_) for the measured distances can be calculated according to the maximum exposure (H_max dose_) using Equation (2). The calculation was based on a peak irradiance (E_irradiance_) of 1.90 µW/cm^2^.

(2) $$H_{\max\;\mathrm{dose}}\;\left[\mathrm{mJ}/\mathrm{cm}²\right]={\;E}_{\mathrm{irradiance}}\;\left[µW/\mathrm{cm}²\right]{\;*\;t}_{\max\;\mathrm{exposure}\;\mathrm{time}}\;\left[s\right]$$

The temperature development during the test was analysed due to the close arrangement of the LEDs around the quartz plate.

***Microbiological testing:*** Test organisms were the following Gram-negative bacteria: *Escherichia coli* (*E. coli* 498 K12), *Acinetobacter kookii* (*A. kookii* DSM 29071), and *Pseudomonas stutzeri* (*P. stutzeri* DSM 4989). The investigated Gram-positive bacteria were *Staphylococcus carnosus* (*S. carnosus* DSM 20501) and *Enterococcus mundtii* (*E. mundtii* DSM 4839). These ESKAPE surrogates were selected on the basis of their demonstrated similar UVC photosensitivities to the pathogenic ESKAPE agents [36]. Additionally, *Micrococcus luteus* (*M. luteus* DSM 20030), as a frequently observed skin dweller, was also included in this study.

The first step involved the preparation of a bacterial suspension. Simultaneously, two quartz plates were prepared and subjected to a thorough cleaning process using 70% ethanol. The plates were then nebulized with a fine mist of the bacterial suspension, resulting in a homogenous bacterial contamination. Afterward, the plates were allowed to dry for 10 min.

The irradiation phase commenced with the initial sampling of both plates: the unirradiated control plate and the plate designated for irradiation. The experimental plate was then exposed to a specific radiation dose for a predetermined period. During the irradiation tests, additional samples were collected from the irradiated plate at defined intervals to monitor bacterial inactivation over time. A final sample was taken from the unirradiated control plate, serving as a control to account for potential experimental artefacts.

For sample collection, the eSwab system from Copan (Brescia, Italy) was used. A volume of 50 µL of eSwab buffer was applied to a 4 × 4 cm^2^ sampling area on the quartz plates, creating five 10 µL droplets, which were pipetted at the corners and the centre of the sampling field (Figure 3). The eSwab was then wiped across the surface for at least 15 s, with a rotational movement counter to the wiping direction, in accordance with the manufacturer’s instructions. The swab was subsequently returned to the tube containing the buffer solution.

Next, the eSwab samples were vortexed for 20 s, a serial dilution was prepared, and the diluted samples were plated on agar plates. After two days of incubation at 37 °C, the bacterial colonies were counted. Throughout the experiment, aseptic techniques were consistently applied. To minimise systematic errors due to potential inhomogeneities in the irradiation, sampling at the various measurement points was performed in a random order.

The experiments were repeated with different strains of bacteria, at least three times for each exposure time. To evaluate the results, a non-linear regression analysis was performed using the GInaFiT Version 1.8 (Geeraerd and Van Impe Inactivation Model Fitting Tool) add-in [37] to investigate the inactivation characteristics of the applied microorganisms.

## 3. Results

***Nebulisation system:*** The nebuliser system achieved uniform distribution with a deviation of approximately 4% in the number of droplets.

The initial CFU concentration of the sprayed cultures showed concentrations in CFU/cm^2^ within the single-digit 10^6^ range after plating and counting. No anomalies or reductions in the bacterial count were detected even after prolonged waiting, meaning that there were no reductions during the course of the test.

**Figure 4:**
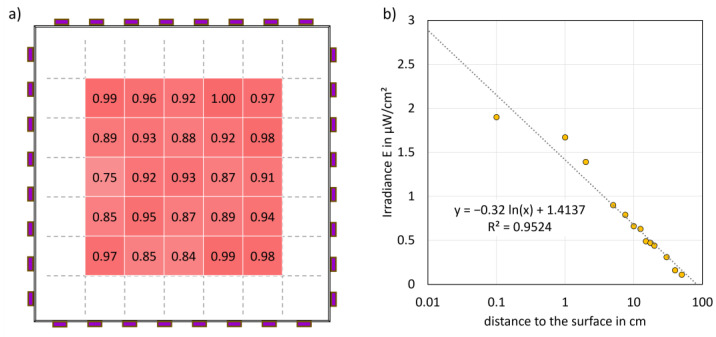
(**a**) Relative homogeneity of the emitted radiation on a large, contaminated quartz glass plate of 30 × 30 cm^2^, with the contaminated sample areas and the normalised measurement points situated within the red area. (**b**) The diagram depicts irradiance as a function of distance to the quartz surface, with a superimposed regression line illustrating the relationship between these variables. The maximum average radiation intensity of 1.90 µW/cm^2^ was measured at a distance of 1 mm.

***Irradiation setup:*** The radiation measurements conducted at a distance of one millimetre demonstrated a predominantly uniform illumination (Figure 4a) of a contaminated glass plate. The highest single irradiance observed was 1.90 µW/cm^2^, whereby the measured values of all replicates had a mean value of 1.09 µW/cm^2^ and a standard deviation of 0.17 µW/cm^2^.

As demonstrated in Equation (3), a stay of up to 26 min at a distance of one millimetre does not exceed the permissible limit value.

(3) $$t_{\max\;\mathrm{exposure}\;\mathrm{time}}=\frac{H_{\max\;\mathrm{dose}}}{E_{\mathrm{irradiance}\;}}=\frac{3\;\mathrm{mJ}/\mathrm{cm}²}{1.90\;µW/\mathrm{cm}²}=1578\;s=26\;\min$$

A further series of measurements conducted from a distance of 1 cm to 50 cm resulted in a maximum irradiance of 0.66 to 0.11 mW/cm^2^ and an exponential fit of the equation E(distance) = −0.32 ln(distance) + 1.4137 µW/cm^2^, with an R^2^ value of 0.95 (Figure 4b).

The quartz plate did not heat up significantly during the test, as the irradiation times were relatively short.

**Figure 5:**
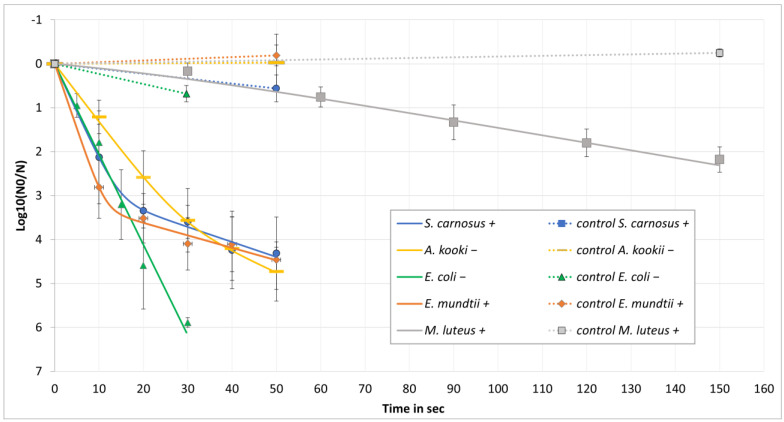
Representation of the decrease in bacterial concentration over time and results of non-linear regression using the GInaFit analysis tool to model behaviour and describe efficacy.

*Microbiological testing:* The photoinactivation of *E. coli*, *A. kookii*, *E. mundtii*, *M. luteus*, and *S. carnosus*, as presented in Figure 5, demonstrated high efficiency when subjected to a disinfection setup using LED irradiation at a current of 40 mA, which is 40% of the maximum LED current. With the exception of *M. luteus*, more than three log reductions in bacterial count were observed within the first 30 s of exposure. *E. coli* exhibited the highest susceptibility to irradiation, achieving a 3 log reduction within just 30 s, characterised by an almost linear decrease in bacterial count in the half logarithmic representation in Figure 5.

The data sets associated with Figure 5 are presented in Table 1 as mean values derived from a minimum of three replicates, including their respective controls and standard deviations.

Table 2 presents the simple linear regression in a semi-logarithmic format, as is also the case in Figure 5, without any form of adjusted modelling. The coefficient of determination R^2^ is a decisive criterion for assessing the quality and fit of regression models. Low R^2^ values indicate that the actual reduction is not adequately represented by a linear fit. Nevertheless, there was a clear difference in the required radiation dose for *M. luteus* compared to the other bacteria. For all three data sets, for which the reduction in the log values was calculated, it could be observed that the necessary reduction doses for *M. luteus* were about ten times higher.

Data associated with Figure 5, including model descriptions and behavioural data, are summarised in Table 3. The modelling therefore offers a superior representation of the reduction behaviour. The highest model quality for *E. coli* was achieved through linear regression, which corroborated the detection limit observed in Figure 5. In contrast, *M. luteus* exhibited a shoulder-+log-linear model, indicative of an extended curve due to its low inactivation rate. For *S. carnosus*, *A. kookii*, and *E. mundtii*, biphasic modelling provided the best fit, revealing that a transition in inactivation behaviour occurred at approximately three log levels. Following this transition, the slope of inactivation became significantly flatter, with variations observed based on the specific organism.

The values for the respective coefficients of determination demonstrate a notable enhancement, which signified an optimised modelling of the reduction behaviour.

All tested organisms were successfully reduced, except for *Pseudomonas stutzeri*, for which no countable CFUs were observed on the quartz plate with or without irradiation.

## 4. Discussion

***Nebulisation system:*** The validation of the nebulisation system demonstrated sufficient accuracy with minimal droplet formation, thereby ensuring largely homogeneous aerosol distribution and preventing the formation of larger droplets due to the hydrophobic surface properties. The bacterial count on the surface was sufficient for the utilisation of the swabs.

***Irradiation setup:*** The distribution of irradiance at a distance of 1 mm revealed that, although the irradiance was irregular, the fluctuations, amounting to only a few µW/cm^2^, are not potentially harmful. A possible issue with the uneven irradiance distribution could be attributed to the simple experimental setup. The 275 nm LEDs with quartz glass optics were not positioned precisely on the lateral surface of the quartz plate, leading to minor angular discrepancies. Given the potential health risks associated with UVC radiation, a peak irradiance of 1.9 µW/cm^2^ and a maximum daily skin exposure of 3 mJ/cm^2^ at a distance of 1 mm were calculated. This upper limit implies that continuous exposure for 26 min is considered safe, provided there is no constant contact with the touchscreen. The functional equation in Figure 4 showed a decrease in irradiance with increasing distance, indicating a reduction in the potential for hazard. It should be noted that the radiation application is not designed to be used for a period of 26 min and that exposure to radiation during interaction with the touchscreen unit is not intended. Once the user is identified, the risk of exposure should be eliminated by switching off.

The brief irradiation times in conjunction with the extensive surface area of the quartz plate concentrate the capabilities of preceding studies [32,33] and demonstrate the applicability of this approach. The applicability of this irradiation method requires quartz glass to ensure the necessary transparency at 275 nm. Unfortunately, quartz glass is brittle and costly, which currently impedes its utilisation for prospective applications [38].

***Microbiological testing*:** This study involved both Gram-positive and Gram-negative bacteria to compare their responses to UVC disinfection. UVC disinfection is widely recognised for its antimicrobial efficacy [27,39,40], as demonstrated by the significant reduction in microorganisms even at low power levels. However, different sensitivities were observed between bacteria on dry, contaminated quartz plates versus liquid cultures [36], with potential causes including bacterial morphology, repair mechanisms, and biochemical composition (proteins, lipids, nucleic acids).

The low R^2^-values for the linear fits in Table 1 for *S. carnosus* and *E. mundtii* indicate that the relationship between irradiation and log reduction is not perfectly linear/exponential. This suggests that additional factors influence the course of the disinfection curve and affect its progression with increasing effectiveness. The GInaFit model analysis provided a more detailed and individualised understanding of disinfection kinetics, demonstrating better accuracy compared to conventional exponential models, with a determination coefficient exceeding 0.95. This model allows for the optimisation of disinfection strategies by accounting for the specific structure and composition of bacteria on dry surfaces.

As previously outlined, *E. coli* was described by a log-linear regression model, which was developed by Bigelow and Esty (1920) [41] and is represented by a simple straight line. This model accurately depicts an exponential decline of the bacterial population. No further influencing factors could be identified subsequently. The modelling of *E. mundtii*, *S. carnosus*, and *A. kooki* was most accurately represented by the Biphasic model with slope tailing, as described by Geeraerd et al. (2000) [42] and Cerf (1977) [43]. The model described here is based on the assumption that the level of UV sensitivity is initially greater and more susceptible to actinic stress. In the subsequent phase, the characteristics undergo a transformation, resulting in the development of heightened resilience to stress. The slope of the curve then begins to decline. In contrast, *M. luteus* is best described by the log-linear + shoulder model, as proposed by Geeraerd et al. (2000) [42] and Mossel et al. (1995) [44]. This model suggests that the onset of bactericidal activity is delayed until the initial factor or mechanism is overcome and the first bacterial reduction occurs.

Notably, the structure of bacterial cell walls seems to influence the UVC sensitivity. Gram-positive bacteria, with their thicker peptidoglycan layer, are generally more resistant, while Gram-negative bacteria, with a thinner peptidoglycan layer and an additional outer membrane, are more susceptible [23,25]. The results, illustrated in Figure 5, show a flatter reduction curve for Gram-positive bacteria (e.g., *S. carnosus* and *E. mundtii*) compared to Gram-negative bacteria (*E. coli* and *A. kookii*) after a 3 log reduction.

Furthermore, the disparate curves of the reduction process suggest the involvement of additional factors influencing the efficacy of the bactericidal effect. It has been demonstrated that pigmentation can influence sensitivity to ultraviolet radiation. Pigmented bacteria absorb more UV radiation, leading to higher doses by similar mortality rates. *E. mundtii* (Figure 5, orange) and *M. luteus* (Figure 5, grey) are confirmed to be the test organisms exhibiting pigmentation [45,46]. The minimal reduction in M. *luteus*, with its pronounced yellow pigment formation, supports this hypothesis. However, *M. luteus* required a three-times-higher log-reduction dose than, e.g., *E. coli* or *Staphylococci*. This is supported by other data on different *Micrococci* [22]. In contrast, *E. mundtii* exhibits a markedly rapid reduction at the outset, despite the presence of pigmentation. In contrast, the Gram-positive *S. carnosus* (Figure 5, blue) was observed to exhibit a similar trend to that of *E. mundtii*, despite the absence of any known pigment formation. This suggests that different repair mechanisms might be present, with varying degrees of importance, and are dependent on the morphology of the organism [45,46,47,48,49,50,51], environmental conditions, and radiation. Consequently, these mechanisms affect the results of irradiation in a manner that varies between microorganisms.

The effectiveness of spraying and drying surfaces was found to be insufficient when *P. stutzeri* was used as a model organism because the bacteria exhibited a high mortality rate during the drying process due to aerobic and dry conditions.

In contrast to conventional cleaning methods, such as chemical cleaning, sequential irradiation provides a more promising solution for preventing contamination. Daily protective layers [31] and thorough cleaning protocols may still carry risks [52], but sequential disinfection methods have the potential to reduce contamination and cross-transfer of microorganisms.

## 5. Conclusions

The conducted experiments have demonstrated the potential of this technology in terms of its effectiveness against various microorganisms for a wide range of applications. Calculations based on the measured irradiation values and defined exposure limits indicate no acute risk when handling UVC radiation. In regard to functional disorder, a continuous skin exposure of 25 min without touching the quartz surface at a distance of 1 mm and interactions totalling up to three and a half minutes of contact time with the touchscreen can be performed without hesitation.

Further studies that include comparisons with other bacterial strains are recommended, as well as with yeast and moulds. Alternative irradiation approaches should also be considered to gain a deeper understanding of microbial behaviour when irradiated on and through dry surfaces.

Irradiating the surface and deactivating the device during use offer an effective disinfection method, reducing microbial load on touchscreens and minimising contamination risks while ensuring user safety from radiation exposure [52,53].

## Figures and Tables

**Table 1:** Irradiation tests results presented in mean log reductions over time, with controls and SDs.

Time in Sec	0	5	10	15	20	30	40	50	60	90	120	150	Control Start	Control End
*S. carnosus +*														
mean	0.00		1.78		2.98	3.65	3.78	3.93					0.00	0.56
SD	0.00		0.79		0.84	0.34	0.71	0.60					0.00	0.30
*E. coli −*														
mean	0.00	0.95	1.79	3.20	4.59	5.89							0.00	0.68
SD	0.00	0.27	0.41	0.79	0.99	0.11							0.00	0.18
*A. kooki −*														
mean	0.00		1.21		2.59	3.56	4.21	4.73					0.00	−0.03
SD	0.00		0.38		0.61	0.73	0.72	0.67					0.00	0.65
*E. mundtii +*														
mean	0.00		2.81		3.52	4.10	4.10	4.46					0.00	−0.19
SD	0.00		0.70		0.56	0.60	0.62	0.29					0.00	0.24
*M. luteus +*														
mean	0.00					0.14			0.70	0.70	1.69	2.09	0.00	−0.25
SD	0.00					0.19			0.23	0.40	0.31	0.29	0.00	0.09

**Table 2:** The resulting times for a 1, 3, and 4 log reduction with linear fit for an assumed exponential decline of the bacterial concentration, together with the associated equation and R^2^.

Strain (Gram)	Linear Fit	R^2^	1 Log Reduction [s]	3 Log Reduction [s]	4 Log Reduction [s]
*S. carnosus +*	y = 0.0973 x	R^2^ = 0.7347	10.28	30.83	41.11
*A. kookii −*	y = 0.1046 x	R^2^ = 0.9576	9.56	28.68	38.24
*E. coli −*	y = 0.2098 x	R^2^ = 0.9843	4.77	14.30	19.07
*E. mundtii +*	y = 0.1106 x	R^2^ = 0.5282	9.04	27.12	36.12
*M. luteus +*	y = 0.0126 x	R^2^ = 0.9089	79.37	238.10	317.46

**Table 3:** Outcomes of the GInaFit adjustment, applied to the respective model type. These include the initial concentration in log_10_, the sum of squared errors (SSE), the root mean-squared error (RMSE), R^2^—the coefficient of determination, and the time for a 4 log-reduction.

Strain (Gram)	Model Type	SSE	RMSE	R^2^	4 Log- Reduction Time [s]
*S. carnosus +*	Biphasic	0.0324	0.1800	0.9938	N/A
*A. kookii −*	Biphasic	0.0080	0.0893	0.9988	46.0
*E. coli −*	Log-Linear	0.0254	0.1594	0.9957	20.1
*E. mundtii +*	Biphasic	0.0461	0.2146	0.9928	39.5
*M. luteus +*	Shoulder + Log-Lin	0.0486	0.2204	0.9584	N/A

## Data Availability

The original contributions presented in the study are included in the article, further inquiries can be directed to the corresponding author.
